# Recent advances in human stem cell-based modeling of Tuberous Sclerosis Complex

**DOI:** 10.1186/s13229-020-0320-2

**Published:** 2020-02-19

**Authors:** Wardiya Afshar Saber, Mustafa Sahin

**Affiliations:** Department of Neurology, Harvard Medical School, Boston Children’s Hospital, 300 Longwood Ave, Boston, MA 02115 USA

**Keywords:** Tuberous sclerosis complex, Autism, Human pluripotent stem cells, CRISPR/Cas9, Neurons, Purkinje neurons, Cortical tuber, Astrocytes, Brain organoids

## Abstract

Tuberous sclerosis complex (TSC) is an autosomal dominant disorder characterized by epilepsy, intellectual disability, and benign tumors of the brain, heart, skin, and kidney. Animal models have contributed to our understanding of normal and abnormal human brain development, but the construction of models that accurately recapitulate a human pathology remains challenging. Recent advances in stem cell biology with the derivation of human-induced pluripotent stem cells (hiPSCs) from somatic cells from patients have opened new avenues to the study of TSC. This approach combined with gene-editing tools such as CRISPR/Cas9 offers the advantage of preserving patient-specific genetic background and the ability to generate isogenic controls by correcting a specific mutation. The patient cell line and the isogenic control can be differentiated into the cell type of interest to model various aspects of TSC. In this review, we discuss the remarkable capacity of these cells to be used as a model for TSC in two- and three-dimensional cultures, the potential variability in iPSC models, and highlight differences between findings reported to date.

## Introduction

### Clinical features

Tuberous sclerosis complex (TSC) is a neurogenetic syndrome with a prevalence of 1 in about 6000 births worldwide [1]. Individuals with TSC are heterozygous for loss-of-function germline mutations in either of the tumor-suppressor genes *TSC1* or *TSC2*, and they can have benign tumors called hamartomas in multiple organs such as the brain, heart, skin, lungs, and kidney [2]. TSC is also associated with neurological impairments including epilepsy, autism spectrum disorder (ASD), attention deficit hyperactivity disorder, and cognitive disabilities [3]. About 25 to 60% of all children with TSC also exhibit ASD and more than 50% have some degree of cognitive impairment [3]. Epilepsy is a major concern in TSC as it can begin in infancy and is medically refractory in about two-thirds of patients. In some cases, surgical resection of the affected brain tissue is able to mitigate the seizure burden. The origins of the neurological symptoms associated with TSC are not well understood. Hallmark pathologies of TSC include cortical tubers, subependymal nodules (SENs), and subependymal giant cell astrocytomas (SEGAs) [4]. Cortical tubers consist of areas of cortical dyslamination containing various cell types such as dysmorphic neurons, giant cells, and reactive astrocytes [5, 6]. Dysmorphic neurons are characterized by abnormal morphology, abnormal orientation, and abnormally large sizes, and their immunophenotype resembles that of cortical projection neurons and suggests an alteration of a selected population of intermediate progenitor cells [7]. Giant cells in tubers have been shown to express proteins that are typically found in immature neurons and immature glia, suggesting a failure to terminally differentiate prior to migration into the cortex [6, 8]. Additionally, clinical manifestations of TSC also include cardiac rhabdomyomas which represent neonatal manifestations of cardiac disease in TSC [9]; renal angiomyolipomas (AMLs) composed of smooth muscle, blood vessels, and adipose tissue; pulmonary and lymphatic manifestations in the form of lymphangioleiomyomatosis (LAM) [10]; and facial angiofibromas and hypomelanotic macules [11]. Rapamycin and its analogues inhibit the activation of the mTOR signaling pathway and have been used to treat patients with TSC. Clinical trials based on rapamycin and its analogues have shown improvement in epilepsy in TSC with 50% seizure reduction in approximatively 40% of individuals [12]. Additionally, rapalogues have also been effective for the treatment of subependymal giant cell astrocytomas (SEGAs), AMLs, and LAM. However, tumors may regrow if treatment is stopped [13]. Neuropsychological deficits and autistic symptoms have also been investigated in clinical trials with rapalogues and have not been as successful as predicted from animal experiments [14, 15]. Therefore, despite some success with rapalogues, there remains unmet clinical needs for TSC treatment [13]. The lack of a detailed understanding of how TSC disease mechanisms affect human neuronal and glial cells, for instance, impairs the development of improved treatment.

### Genetics

TSC can be inherited in an autosomal dominant manner, with clinical features varying widely between individuals. Approximately one-third of individuals with TSC have inherited a *TSC1* or a *TSC2* mutation while two-thirds of cases arise from de novo germline mutations [2]. Additionally, many cases result from genetic mosaicism in which a somatic mutation in *TSC1* or *TSC2* occurs during early embryonic development [16]. The somatic inactivation of the wild-type alleles of *TSC1* and *TSC2* can be explained by several possible mechanisms such as loss of heterozygosity (LOH), mutation, and promoter methylation [17]. TSC1 and TSC2 respectively encode for the proteins hamartin and tuberin, which together negatively regulate the mechanistic target of rapamycin complex 1 (mTORC1) [18]. mTORC1 is a kinase that regulates cell growth and anabolic processes in response to amino acids, stress, oxygen, energy, and growth factor stimulation and is acutely sensitive to rapamycin. TSC exhibits a high variability in the phenotypic expression such as the symptoms, age of onset, and severity of the disease [19]. For instance, pathological lesions including cortical tubers, the hallmark finding in TSC, are variable and appear stochastically. Additionally, tumor development in TSC fits the Knudson two-hit tumor-suppressor gene model with a second hit event causing the inactivation of the remaining wild-type allele of either *TSC1* or *TSC2* [20]. This heterogeneity arises from stochastic factors that affect the number and distribution of these second hits but also possibly from cell-specific mechanisms in response to the mutation and mosaicism. The phenotypic heterogeneity poses major challenges in the development of models to recapitulate the full pathology seen in human TSC and identifying effective treatments for TSC. Both patient-specific genetic background and somatic mutations in different tissues together contribute to the complex genetic tapestry underlying TSC disease. Therefore, the iPSCs generated from two different somatic cells from the same individual may carry somewhat distinct genetic background. To overcome these shortcomings, it is crucial to use isogenic controls (in which a mutation has been corrected in an iPSC clone) as much as possible. To obtain reproducible and generalizable results, it will also be important to test more than one line from each patient and several patients with different *TSC1* or *TSC2* mutations.

While most of the studies have been focusing on the cell-autonomous effect of mTORC1 in TSC1- or TSC2-deficient cells, less is known about the non-cell-autonomous effect of TSC1/2-deficiency on the microenvironment. Non-cell-autonomous effects of TSC1/2 loss represent an emerging area of investigation; for example, we reported effects of *Tsc1* deletion resulting in an increase in connective tissue growth factor (CTGF) secretion that non-cell autonomously stunts oligodendrocyte development [21]. Studies also report the effects of TSC2-deficient cells on neighboring wild-type cells, lymphatic endothelial cells, and inflammatory cells and pathways in the brain and in tumors [22].

## Human cellular experimental models of TSC

**Fig. 1:**
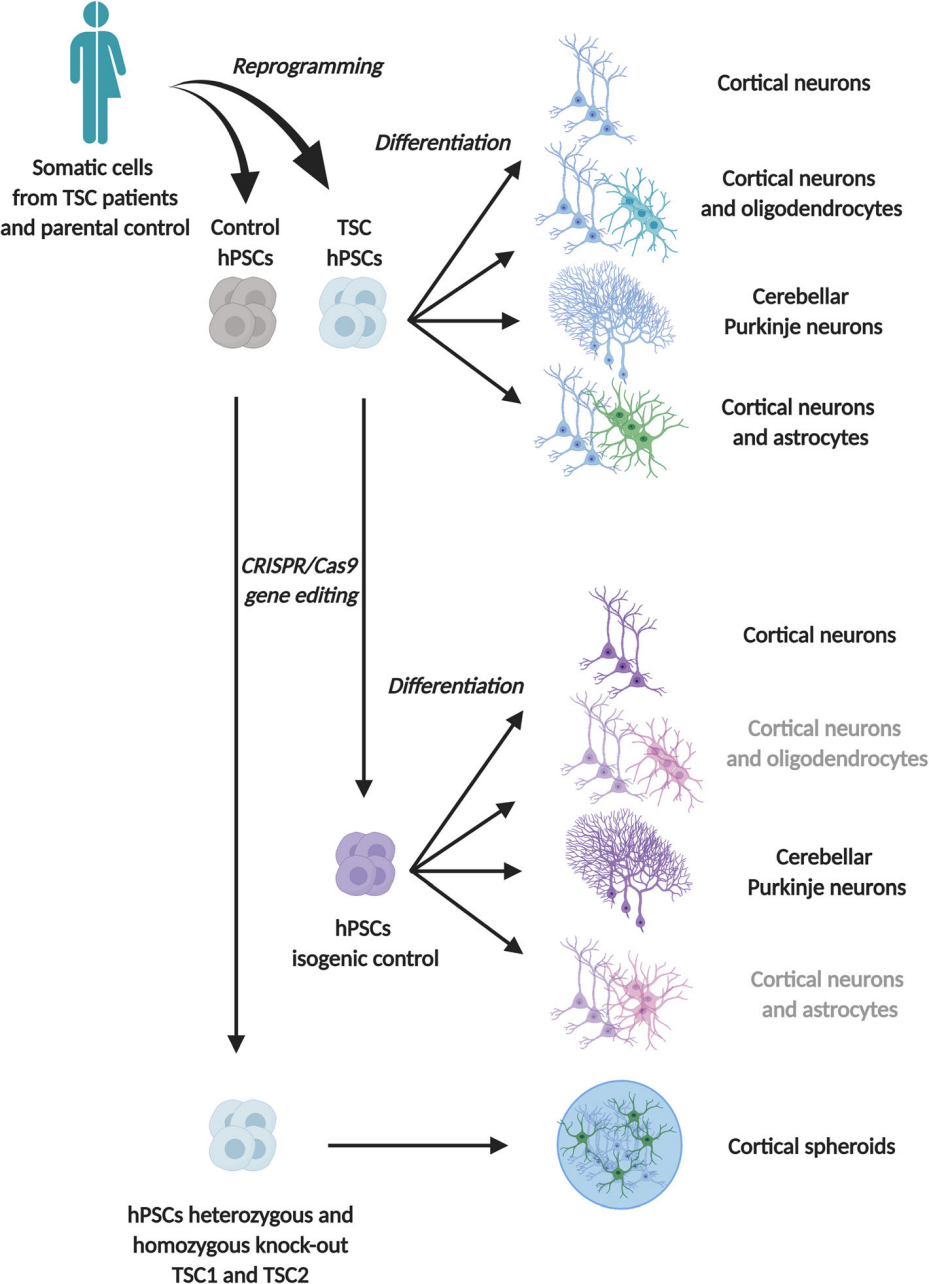
Human neuronal models of TSC. Various approaches to generate cellular models of TSC with pluripotent stem cells. Somatic cells from TSC patients and parental control can be reprogrammed into pluripotent stem cells and differentiated in the cell type of interest to model neurological aspects of TSC. In gray, models that have not been yet published using isogenic controls

Rodent models have contributed to key discoveries with regard to the consequences of TSC1 and TSC2 loss on brain development and function, including that complete loss of *Tsc1* or *Tsc2* in germline knockout mouse models causes embryonic lethality prior to brain development. This limitation impedes the study of cortical tubers and the earliest stages of neural development in rodent models. Additionally, heterozygous animals have subtle phenotypes whereas the TSC patients are heterozygous. These findings demonstrate that there are important differences between animal models and the human phenotype. Therefore, human cellular models are necessary to study how alterations in TSC-mTOR signaling affect these features. Recent advances with the derivation of hiPSCs from skin or blood cells from patients have opened new avenues to the study of TSC [23] (Table 1). This approach combined with gene-editing tools such as CRISPR/Cas9 offers the advantage of preserving patient-specific genetic and generating isogenic controls by correcting a specific mutation [31]. The patient cell line and the isogenic control can be differentiated into the cell type of interest to model various aspects of TSC, including neurons and astrocytes (Fig. 1).

**Table 1:** Recapitulative table of human neuronal models of TSC

Source	Genotype	Control	Cells generated	Model	Main findings	Treatment
Fibroblasts	TSC1^+/−^ TSC2^+/−^	Familial	Cortical neurons and oligodendrocytes (OL) [24].	2D	Increased network activity, cellular hypertrophy, augmentation of OL proliferation and decrease of OL maturation [24].	Rapamycin and guanabenz improved the reduced maturation observed in TSC neuron-OL co-cultures [24]. Only rapamycin showed regulating effects on soma size when co-cultures contained TSC neurons and/or TSC OLs [24].
Fibroblasts and peripheral blood mononuclear cells	TSC2^+/−^ TSC2^−/−^	Familial and CRISPR/Cas9	Cerebellar Purkinje neurons [25]	2D	Reduced synaptic activity, hypoexcitability, mTORC1 pathway hyperactivation [25].	Rapamycin treatment rescued the deficits in differentiation, synaptic dysfunction, and hypoexcitability of TSC2 mutant hiPSC-PCs in vitro [25].
Peripheral blood mononuclear cells	TSC2^+/−^ TSC2^−/−^	Familial and CRISPR/Cas9	Cortical neurons co-culture with wild-type astrocytes [26]	2D	Loss of one allele of TSC2 is sufficient to cause some morphological and physiological changes in human neurons [26]. Biallelic mutations in TSC2 are necessary to induce gene expression dysregulation present in cortical tubers [26].	Rapamycin treatment reduced neuronal activity and partially reversed gene expression abnormalities [26].
Peripheral blood mononuclear cells	TSC2^+/−^	Familial	Neurons and astrocytes [27]	2D	Enlargement of the soma, perturbed neurite outgrowth, and abnormal connections among cells [27]. Increased saturation density and higher proliferative activity in astrocytes [27].	Rapamycin treatment decreased proliferation [27].
Peripheral blood mononuclear cells	TSC2^+/−^	Familial	Neurons [28]	2D	Delayed in their ability to differentiate into neurons [28]. Heterozygous TSC2 mutations disrupt neuronal development potentially due to dysregulated PI3K/AKT signaling [28].	Rapamycin analogue (RAD001) treatment failed to correct the neuronal differentiation defect in patient cells and did not alter the differentiation of control cells [28]. AKT inhibitor (MK2206) and PI3K inhibitor (LY294002) treatments significantly reduced the fraction of HuC/D+ cells in control cultures derived from both unaffected individuals, mimicking the phenotype of TSC2 haploinsufficient cell lines [28].
Gene editing in human embryonic stem cells	TSC2^+/−^ TSC2^−/−^	Heterozygous and homozygous deletions of TSC2	Neurons [29]	2D	Gene-dosage-dependent mTORC1 hyperactivity in neurodevelopment [29]. Altered synaptic transmission paralleled by molecular changes in pathways associated with autism [29].	Rapamycin treatment at different developmental stages suggests that the neurodevelopment and synaptogenesis can be uncoupled and corrected independently of each other [29].
Gene editing in human embryonic stem cells	TSC1^+/−^ TSC1^−/−^ TSC2^+/−^ TSC2^−/−^	CRISPR/Cas9	Cortical spheroids [30]	3D	Mosaic biallelic inactivation during neural progenitor expansion is necessary for the formation of dysplastic cells and increased glia production [30].	Rapamycin treatment results suggest that there is a developmental window for pharmacological mTORC1 suppression to prevent neuronal differentiation defects caused by loss of TSC2. Later rapamycin treatment cannot reverse cell fate decisions that have already been made but can rescue mTORC1 hyperactivation and reduce neuronal and glial hypertrophy. Sustained mTORC1 inhibition is required to prevent the re-emergence of mTORC1 hyperactivity in differentiated cells [30].

### In vitro model for cortical phenotypes

The lack of a detailed understanding of how TSC disease mechanisms affect human neurons and glial cells has been an obstacle to the development of improved treatments. While allowing the study of human-specific biology, the use of human stem cells enables the study of the early stages of neural development relevant for TSC. For instance, cortical excitatory neurons and astrocytes of the telencephalic lineage can be generated through manipulation of endogenous neuroectodermal differentiation pathways via either inhibition of the dual-SMAD pathway [32] or exogenous expression of transcription factors [33] to investigate the formation of cortical tubers in TSC (Fig. 1). Genetically engineered human embryonic stem cells [29], TSC patient-derived iPSCs [24, 27, 28], and gene-edited TSC iPSCs [34] have been generated and differentiated into neural progenitor cells (NPCs), neurons, astrocytes, and oligodendrocytes [24], using various differentiation protocols to investigate the role of TSC1 and TSC2. TSC2^−/−^ cultures exhibited an increase in neural rosette size and produced significantly lower numbers of cells expressing the neuronal markers HuC/D [29] (Table 1). Additionally, TSC2^−/−^ neurons displayed increased dendritic arborization while NPCs, neurons, and glia exhibited somatic hypertrophy [29]. In contrast, TSC2^+/−^ cultures exhibited an increased proliferation rate in some studies [27] and not others [28] (Table 1). Interestingly, cultures of cells with heterozygous *TSC1* or *TSC2* loss exhibited either a minor decrease in HuC/D-positive cells [28, 29] or no decrease [24]. Contradictory findings have also been published with no change in neuronal morphology [28, 29], minor increases in dendritic branching and no change in the soma size [24], or increases in both [27]. Functional studies were performed to identify electrophysiological phenotypes and showed reduced intrinsic excitability in TSC2^−/−^ but not in TSC2^+/−^ neurons which supports the observations of the change in morphology [29] (Fig. 1). Furthermore, a decrease in the frequency of excitatory postsynaptic currents was observed in TSC2^+/−^ and TSC2^−/−^ neurons in a gene dose-dependent manner [29] (Table 1). In contrast, multi-electrode array recordings of heterozygous iPSC-derived neurons exhibited an increase in spontaneous network activity [24]. Furthermore, calcium imaging in these cultures revealed increased frequency but not amplitude [24] (Fig. 1). Discrepancies between the findings may reflect gene dose-dependent effects of cell lines and culture variability. Moreover, transcriptomic analysis of isogenic, gene-edited TSC2 heterozygous and homozygous cultures showed significant differences between TSC2^−/−^ and TSC2^+/+^ neurons but not between TSC2^+/−^ and TSC2^+/+^ neurons [26, 35]. These phenotypic differences need to be further investigated with additional iPSC-derived TSC patients and control cell lines to determine the link to the *TSC2* mutation, cell line variability, or genetic background (Fig. 1). Additionally, although increased levels of phospho-S6 and increased cell growth as a consequence of the hyperactivation of mTORC1 was a common result of all studies, the strong effects seen at every developmental stage in TSC2^−/−^ cultures [29] were not consistently seen at the NPC stage in TSC2^+/−^ cultures [28, 30]. Finally, treatment with rapalogues and other mTOR inhibitors rescued the altered phenotypes previously described in the TSC1 or TSC2 loss in forebrain neural cultures [24, 26–29].

### Isogenic systems

While forebrain excitatory neurons offer the advantage to study tuber formation, cerebellar Purkinje cells have been demonstrated to be relevant to TSC pathophysiology, particularly the behavioral symptoms of autism [36–38]. In order to establish a cerebellar model to study TSC, a differentiation protocol was successfully developed and used to differentiate hiPSC lines from three individuals with TSC into cerebellar Purkinje cells (Fig. 1) [25]. Additionally, in this study, CRISPR/Cas9 was used to create a *TSC2*^*−/−*^ cell line together with a repaired *TSC2*^*+/+*^ control cell line, which provides an isogenic system [34] (Table 1). Isogenic systems have the advantage to be based on cells with the same genetic background, except for the gene of interest making the model an ideal control. This study has demonstrated similar phenotypes as the forebrain cultures in both heterozygous and homozygous cultures, with more severe deficiencies in TSC2^−/−^ cells such as increased rates in NPCs proliferation, increased cell growth, hyperactivation of mTORC1 activity, and hypoexcitability of differentiated cerebellar Purkinje neurons (Fig. 1) [25]. Importantly, this hypoexcitability confirmed previous findings from the Purkinje cell-specific mouse model [36]. Similar to the forebrain neurons, RNA sequencing revealed more differential gene expression between TSC2^−/−^ and TSC2^+/+^ than TSC2^+/−^ and TSC2^+/+^ Purkinje neurons. Finally, treatment with mTOR inhibitors reversed all of the observed phenotypic effects of complete TSC2 loss [25].

### Three-dimensional models

While the differentiation protocols used to generate the models previously described have been conducted in two-dimensional cultures (2D), recent advances in three-dimensional (3D) differentiation techniques to generate human stem cell-derived brain organoids provide a new platform to investigate neurodevelopmental disorders [39, 40]. These 3D models recapitulate many developmental processes of the human brain, including progenitor zones and rudimentary cortical layers [41], which could provide new insight to the study of the cortical tubers in TSC considering that these developmental malformations are linked to altered differentiation and defective migration (Fig. 1). Recently, an interesting approach was taken in a study combining human brain organoids and CRISPR/Cas9 as a means to investigate the “two-hit” hypothesis of cortical tuber development [30] (Fig. 1). Consistent with results from 2D neuronal cultures, a strong bias towards an astroglial cell fate, altered cell morphology, and activation of mTORC1 signaling were observed in this model [30, 42] (Table 1). Additionally, it was shown that mosaic biallelic inactivation during neural progenitor expansion is necessary for the formation of dysplastic cells and increased glia production in three-dimensional cortical spheroids [30]. Furthermore, while it has been suggested that prenatal rapalogue treatment could be beneficial to prevent developmental abnormalities in TSC [43], this study shows that strong mTORC1 suppression during early development can alter the normal pattern of cortical differentiation [30]. Moreover, removal of rapamycin after early treatment caused the return of mTORC1 hyperactivity in *TSC2* KO cells, indicating the potential necessity of chronic rapalogue use to fully treat TSC-associated phenotypes in culture [30].

Collectively, the studies described in this section demonstrate the relevance and the potential of human stem cell-based modeling of neurodevelopmental disorders such as TSC, which could facilitate further testing of therapeutics and identify critical developmental windows for treatment. However, clinical manifestations of TSC also include renal angiomyolipomas (AMLs), cardiac rhabdomyomas, and lymphangioleiomyomatosis (LAM). The phenotypes resulting from the loss of TSC1 or TSC2 can vary across all stages of development and are likely lineage-dependent. Therefore, the development of better tumor models of TSC to investigate human AML or LAM would greatly contribute to our understanding of TSC etiology.

### Tumor models for AML and LAM

Lymphangioleiomyomatosis occurs only in post-pubescent females, has a median age of diagnosis of 35, and affects 30% of female TSC patients [44]. LAM is defined by acquisition of inactivating mutations in one of two tumor-suppressor genes *TSC1* or *TSC2* [44]. LAM is characterized by pulmonary infiltration of abnormal smooth muscle-like cells that cause cystic replacement of the lung parenchyma, progressive tissue destruction, and ultimately respiratory failure [22]. Major limitations, such as the inability to propagate patient-derived TSC1/2-deficient LAM cells in culture without immortalization, impair the development of an appropriate human cellular model. In fact, cultures of cells derived from LAM tumor biopsies grow as a heterogeneous population of TSC2^+/+^ and TSC2^−/−^ cells with increased activation of mTOR, and currently, there is no homogeneous clonal population of TSC2^−/−^ pulmonary cells which has been established [45]. An attempt to reprogram LAM lung cells derived from transplant resulted in hiPSC lines that exhibited normal TSC2 and TSC1 expression [44]. LAM lung cells, as defined by *TSC2* mutation and loss of heterozygosity, do not seem to grow as a clonal population in cell culture; these cells are only detected in the presence of TSC2 wild-type cells after enrichment. To overcome this limitation, the fact that LAM lesions are comprised of cells that express markers of the neural crest cell (NCC) lineage, including expression of smooth muscle cell (SMC) markers, suggests an NCC-SMC origin. Therefore, a novel cell model of LAM using a patient cell reprogramming approach was developed focusing on the rationale that LAM cells arise from TSC1/2-deficient cells within the SMC lineage. These human mesenchymal models of TSC recapitulate multiple aspects of TSC tumors, but the origin of the mesenchymal features of TSC is less clear. Very recently, a human pluripotent stem cell-based model of the multi-lineage manifestations of TSC has been developed [46]. The approach taken for this study was based on the stem cell-like qualities of NCCs, thus providing the possibility to model multiple aspects of mesenchymal TSC tumors in a progenitor cell lineage. CRISPR/Cas9 was used to introduce an inactivating mutation in the TSC2 locus of four hPSC lines for the generation of either NPCs or NCCs. In this study, TSC2^−/−^ NPCs and neuronal and glial derivatives accurately model critical features of neurological TSC tumors as well as the TSC2^−/−^ NCCs for mesenchymal TSC tumors [46]. Importantly, this study revealed that TSC2^−/−^ NPCs are selectively sensitized to proteasome inhibition with clinically relevant compounds, in the absence of mTORC1 inhibition with rapamycin, suggesting this therapeutic approach holds promise as a stand-alone therapy or complimentary treatment to existing regimens for the neurological, but not mesenchymal, features of TSC [46]. These results highlight the strength of a multisystem hPSC modeling approach as it could reveal key lineage-specific mechanisms in TSC and potentially enable the development of improved treatments.

Angiomyolipomas (AMLs) are tumors composed of smooth muscle, blood vessels, and adipose tissue. Malignant forms of AMLs have been reported in patients with TSC, and the cell of origin of AMLs is unknown [47]. AML cells show loss of heterozygosity for either *TSC1* or *TSC2* [48] resulting in the overactivation of the mTORC1 pathway, AML cell growth, and increased production of vascular endothelial growth factor D (VEGF-D) which enables the AML to maintain its nutrition as it enlarges [49]. These tumors have been used to develop cell lines that can serve as models for LAM, since it is difficult to establish cell lines from pulmonary LAM cells as previously described [44, 45]. The LAM patient-associated angiomyolipoma-derived 621–101 cells have been used to elucidate the role of estrogens [50, 51], prostaglandins [52], and autophagy [53]. Additionally, cells isolated from AMLs from female and male patients with TSC expressed CD44v6 and have been shown to require epidermal growth factor (EGF) to grow [54, 55]. Although these models have contributed to the improvement of our understanding of TSC pathogenesis with progress in clinical and translational research in the development of FDA-approved agents for the treatment of AML, SEGAs, and LAM, important gaps and questions remain, particularly involving the neurological manifestations of TSC [56]. Furthermore, there is still no human model to investigate several aspects of TSC such as rhabdomyomas which impairs the development of improved treatment for TSC.

## Conclusions

The clinical features of TSC are highly variable even among patients with identical gene mutations. The generation of hiPSCs from TSC patients enables studies on human models, thus offering the opportunity to answer questions about the basic function of TSC1 and TSC2 in multiple developing tissue types while addressing genotype-phenotype correlations and potential modifiers. However, while human stem cell-based models offer new avenues for the study of TSC, the variability and stochasticity with which different cell types are generated is a potential impediment to reproducibility. Additionally, important caveats must be considered when using hiPSCs to model neurological aspects such as the maturity of the cells generated. This feature could be the opportunity to study abnormalities related to the brain development of TSC patients; however, it can present a challenge to study aspects of TSC that may emerge later in development. Additionally, neuronal differentiation protocols have been developed to generate specific neural cell types arising from a specific developmental lineage. Therefore, it is essential to consider what cell types are most relevant to the study of TSC. While offering the advantage of preserving patient-specific genetic mutation, a major challenge for human stem cell-based disease modeling resides in establishing an appropriate control. For instance, using cell lines generated from different individuals could reflect cell line variability or differences in genetic background unrelated to disease state. Fortunately, the emergence of new technologies for gene editing such as CRISPR/Cas9 overcome this limitation by facilitating the generation of isogenic cell lines. Furthermore, recent advances in gene editing can also be used for the expression of additional tools such as optogenetic proteins for neuronal activation or silencing and genetically encoded calcium or voltage indicators to monitor neuronal activity [57–59]. Taken together, the combination of these emerging technologies can facilitate the development of human models of TSC to potentially reveal key mechanisms of the disease and give insights into treatments to contribute to advances in the field.

## Data Availability

Not applicable

## Abbreviations

2D: Two-dimensional
3D: Three-dimensional
AMLs: Renal angiomyolipomas
ASD: Autism spectrum disorder
CTGF: Connective tissue growth factor
EGF: Epidermal growth factor
hiPSCs: Human-induced pluripotent stem cells
LAM: Lymphangioleiomyomatosis
LOH: Loss of heterozygosity
mTORC1: Mechanistic target of rapamycin complex 1
NCC: Neural crest cell
NPC: Neural progenitor cell
OL: Oligodendrocytes
SEGAs: Subependymal giant cell astrocytomas
SENs: Subependymal nodules
SMC: Smooth muscle cell
TSC: Tuberous sclerosis complex
VEGF-D: Vascular endothelial growth factor D

## References

[1] Osborne JP, Fryer A, Webb D (1991). Epidemiology of tuberous sclerosis. Ann N Y Acad Sci.

[2] Curatolo Paolo, Bombardieri Roberta (2007). Tuberous sclerosis. Malformations of the Nervous System.

[3] Crino PB, Nathanson KL, Henske EP (2006). The tuberous sclerosis complex. N E J Med.

[4] Katz JS (2017). Unique findings of subependymal giant cell astrocytoma within cortical tubers in patients with tuberous sclerosis complex: a histopathological evaluation. Childs Nerv Syst.

[5] Mizuguchi M, Takashima S (2001). Neuropathology of tuberous sclerosis. Brain Dev.

[6] Boer K (2009). Doublecortin-like (DCL) expression in focal cortical dysplasia and cortical tubers. Epilepsia.

[7] Mühlebner A (2016). Specific pattern of maturation and differentiation in the formation of cortical tubers in tuberous sclerosis complex (TSC): evidence from layer-specific marker expression. J Neurodev Dis.

[8] Talos DM (2008). Cell-specific alterations of glutamate receptor expression in tuberous sclerosis complex cortical tubers. Ann Neurol.

[9] Watson GH (1991). Cardiac rhabdomyomas in tuberous sclerosis. Ann N Y Acad Sci.

[10] Glasgow CG (2008). Lymphatic involvement in lymphangioleiomyomatosis. Ann N Y Acad Sci.

[11] Volpi A (2019). Tuberous sclerosis complex: new insights into clinical and therapeutic approach. J Nephrol.

[12] Overwater IE (2019). Everolimus for the treatment of refractory seizures associated with tuberous sclerosis complex (TSC): current perspectives. Ther Clin Risk Manag.

[13] Habib SL (2016). Is mTOR inhibitor good enough for treatment all tumors in TSC patients?. J Cancer.

[14] Krueger DA (2017). Everolimus for treatment of tuberous sclerosis complex-associated neuropsychiatric disorders. Ann Clin Transl Neurol.

[15] Overwater IE (2019). A randomized controlled trial with everolimus for IQ and autism in tuberous sclerosis complex. Neurology.

[16] Giannikou K, et al. Low-level mosaicism in tuberous sclerosis complex: prevalence, clinical features, and risk of disease transmission. Genet Med. 2019. 10.1038/s41436-019-0562-6 31160751

[17] Martin KR (2017). The genomic landscape of tuberous sclerosis complex. Nat Commun.

[18] Laplante M, Sabatini DM (2012). mTOR signaling in growth control and disease. Cell.

[19] Kohrman MH (2012). Emerging treatments in the management of tuberous sclerosis complex. Pediatric Neurol.

[20] Knudson AG (1971). Mutation and cancer: statistical study of retinoblastoma. Proc Natl Acad Sci U S A.

[21] Ercan E (2017). Neuronal CTGF/CCN2 negatively regulates myelination in a mouse model of tuberous sclerosis complex. J Exp Med.

[22] Henske EP (2016). Tuberous sclerosis complex. Nat Rev Dis Prim.

[23] Takahashi K (2007). Induction of pluripotent stem cells from adult human fibroblasts by defined factors. Cell.

[24] Nadadhur AG (2019). Neuron-glia interactions increase neuronal phenotypes in tuberous sclerosis complex patient iPSC-derived models. Stem Cell Rep.

[25] Sundberg M (2018). Purkinje cells derived from TSC patients display hypoexcitability and synaptic deficits associated with reduced FMRP levels and reversed by rapamycin. Mol Psychiatr.

[26] Winden KD (2019). Biallelic mutations in TSC2 lead to abnormalities associated with cortical tubers in human iPSC-derived neurons. J Neurosci.

[27] Li Y (2017). Abnormal neural progenitor cells differentiated from induced pluripotent stem cells partially mimicked development of TSC2 neurological abnormalities. Stem Cell Rep.

[28] Zucco AJ (2018). Neural progenitors derived from tuberous sclerosis complex patients exhibit attenuated PI3K/AKT signaling and delayed neuronal differentiation. Mol Cell Neurosci.

[29] Costa V (2016). mTORC1 inhibition corrects neurodevelopmental and synaptic alterations in a human stem cell model of tuberous sclerosis. Cell Reports.

[30] Blair JD, Hockemeyer D, Bateup HS (2018). Genetically engineered human cortical spheroid models of tuberous sclerosis. Nat Med.

[31] Giacalone JC (2018). CRISPR-Cas9-based genome editing of human induced pluripotent stem cells. Curr Protoc Stem Cell Biol.

[32] Chambers SM (2009). Highly efficient neural conversion of human ES and iPS cells by dual inhibition of SMAD signaling. Nat Biotechnol.

[33] Zhang Y (2013). Rapid single-step induction of functional neurons from human pluripotent stem cells. Neuron.

[34] Ebrahimi-Fakhari D (2016). Impaired mitochondrial dynamics and mitophagy in neuronal models of tuberous sclerosis complex. Cell Rep.

[35] Grabole N (2016). Genomic analysis of the molecular neuropathology of tuberous sclerosis using a human stem cell model. Genome Med.

[36] Tsai PT (2012). Autistic-like behaviour and cerebellar dysfunction in Purkinje cell Tsc1 mutant mice. Nature.

[37] Stoodley CJ (2017). Altered cerebellar connectivity in autism and cerebellar-mediated rescue of autism-related behaviors in mice. Nat Neurosci.

[38] Tsai PT (2018). Sensitive periods for cerebellar-mediated autistic-like behaviors. Cell Rep.

[39] Kadoshima T (2013). Self-organization of axial polarity, inside-out layer pattern, and species-specific progenitor dynamics in human ES cell-derived neocortex. Proc Natl Acad Sci U S A.

[40] Paşca AM (2015). Functional cortical neurons and astrocytes from human pluripotent stem cells in 3D culture. Nat Methods.

[41] Bershteyn M (2017). Human iPSC-derived cerebral organoids model cellular features of lissencephaly and reveal prolonged mitosis of outer radial glia. Cell Stem Cell.

[42] Blair JD, Bateup HS. New frontiers in modeling tuberous sclerosis with human stem cell-derived neurons and brain organoids. Developmental Dynamics. 2019. 10.1002/dvdy.60 PMC6995669 31070828

[43] Magri L, Galli R (2013). mTOR signaling in neural stem cells: from basic biology to disease. Cell Mol Life Sci.

[44] Julian LM (2017). Human pluripotent stem cell-derived TSC2-haploinsufficient smooth muscle cells recapitulate features of lymphangioleiomyomatosis. Cancer Res.

[45] Krymskaya VP (2008). Smooth muscle-like cells in pulmonary lymphangioleiomyomatosis. Proc Am Thorac Soc.

[46] Delaney SP, et al. Stem cell models identify lineage-specific catabolic signaling, neoplastic mechanisms and therapeutic vulnerabilities in tuberous sclerosis. bioRxiv. 2019:683359.

[47] Al-Saleem T (1998). Malignant tumors of the kidney, brain, and soft tissues in children and young adults with the tuberous sclerosis complex. Cancer.

[48] Henske EP (1995). Loss of heterozygosity in the tuberous sclerosis (TSC2) region of chromosome band l6p13 occurs in sporadic as well as TSC-associated renal angiomyolipomas. Genes Chromosomes Cancer.

[49] El-Hashemite N (2003). Mutation in TSC2 and activation of mammalian target of rapamycin signalling pathway in renal angiomyolipoma. Lancet.

[50] Yu J, Henske EP (2010). mTOR activation, lymphangiogenesis, and estrogen-mediated cell survival: the “perfect storm” of pro-metastatic factors in LAM pathogenesis. Lymphat Res Biol.

[51] Yu J (2004). Estradiol and tamoxifen stimulate LAM-associated angiomyolipoma cell growth and activate both genomic and nongenomic signaling pathways. Am J Physiol Lung Cell Mol Physiol.

[52] Steagall WK (2013). Osteoprotegerin contributes to the metastatic potential of cells with a dysfunctional TSC2 tumor-suppressor gene. Am J Pathol.

[53] Li C (2016). Proapoptotic protein Bim attenuates estrogen-enhanced survival in lymphangioleiomyomatosis. JCI Insight.

[54] Lesma E (2009). The methylation of the TSC2 promoter underlies the abnormal growth of TSC2 angiomyolipoma-derived smooth muscle cells. Am J Pathol.

[55] Lesma E (2005). Isolation and growth of smooth muscle-like cells derived from tuberous sclerosis complex-2 human renal angiomyolipoma: epidermal growth factor is the required growth factor. Am J Pathol.

[56] Sahin M (2016). Advances and future directions for tuberous sclerosis complex research: recommendations from the 2015 strategic planning conference. Pediatric Neurol.

[57] Kiskinis E (2018). All-optical electrophysiology for high-throughput functional characterization of a human iPSC-derived motor neuron model of ALS. Stem Cell Rep.

[58] Afshar Saber W (2018). All-optical assay to study biological neural networks. Front Neurosci.

[59] Roberts B (2017). Systematic gene tagging using CRISPR/Cas9 in human stem cells to illuminate cell organization. Mol Biol Cell.

